# Congenital Heart Disease and Its Impact on the Development of Anastomotic Strictures after Reconstruction of Esophageal Atresia

**DOI:** 10.1155/2018/6021014

**Published:** 2018-05-20

**Authors:** Pernilla Stenström, Martin Salö, Magnus Anderberg, Einar Arnbjörnsson

**Affiliations:** Department of Pediatric Surgery, Lund University and Skåne University Hospital, Lund, Sweden

## Abstract

**Background:**

The aim was to explore if severe congenital heart disease (CHD) influenced the need for dilatation of anastomotic strictures (AS) after the repair of esophageal atresia (EA).

**Methods:**

A retrospective case-control study was conducted examining AS in children with EA and Gross type C. The spectra of CHD and cardiac interventions were reviewed. The frequency of dilatations of AS during the first year following EA reconstruction was compared between children with and without severe CHD requiring cardiac surgery during their first year of life. Endoscopic signs of stricture were an indication for dilatation.

**Results:**

Included in the follow-up for AS were 94 patients who had EA reconstructions, of whom 10 (11%) children had severe CHD requiring surgery during the first year including 19 different cardiac interventions. In total, 38 patients needed dilatation of esophageal AS, distributed as six (60%) with severe CHD and 32 (38%) without severe CHD (*p* = 0.31).

**Conclusion:**

Severe CHD was present in 11% of children with EA. Esophageal AS developed in 60% children with concomitant CHD, but although high, it did not reach statistical difference from children without CHD (38%).

## 1. Introduction

Congenital heart disease (CHD) constitutes a spectra of cardiac malformations reported to occur in one-third of children with esophageal atresia (EA) [1]. Severe types of CHD are reported to be present in 10% of patients with EA and seem to impact on their survival [1, 2]. Anastomotic strictures (AS) reportedly occur in 9–79% [3–8] of newborns after reconstruction of EA, and hypo-oxygenation of the tissue has been recognized to be one risk factor for development of AS [3]. Since severe CHD often goes with low tissue oxygenation, children with CHD may hypothetically be more prone to develop a stricture at the site of EA anastomosis. However, although the postoperative course after EA reconstruction has been evaluated with regard to risk factors and AS [9–11], the possible influence of CHD on the postoperative development of AS has not been addressed previously. This knowledge could be useful in predicting the postoperative course and planning the medical care after EA reconstructions in children with concomitant CHD.

The aim of the study was to analyze the hypothesis of the impact of severe CHD on need for dilatation of anastomotic stricture after repair of EA.

## 2. Patients and Methods

Children with EA Gross type C who underwent surgical repair between January of 1996 and May of 2016 at a tertiary pediatric surgery center were included in the cohort. This children's hospital houses one of the country's two national specialized centers in medicine (NSCM) for pediatric cardiac surgery. The center for pediatric surgery and center for pediatric cardiac surgery serve as catchment areas for 2 million and 5 million residents, respectively. All children with concomitant EA and severe CHD undergo EA reconstruction at the hospital.

All patients with EA underwent echocardiography prior to EA repair. CHD was divided into minor CHD and severe CHD if cardiac surgical repair was needed during the first year of life.

Children with EA and CHD requiring cardiac surgery at any time during the first year were selected and compared with children with EA without CHD in need of cardiac surgery. Beginning in 2009, patients with EA were registered in a prospective register that was analyzed retrospectively. Patients treated before 2009 had their data compiled retrospectively from their medical records. During the study period, the surgical repairs of EA were all performed by a group of seven pediatric surgeons who had special training in treating EA malformations.

The type of esophageal atresia repair of the children with EA included was the same during the study period. The operation was performed through a posterolateral extrapleural right-sided thoracotomy. The azygous vein was exposed and ligated. The tracheo esophageal fistula (TEF) was identified and closed with two absorbable stiches. The upper esophageal pouch was mobilized from the proximal trachea. Anastomose with a single layer 5/0 sutures was performed and a nasogastric transanastomotic feeding tube, 6–8 French, was left. The latter was used for postoperative feeding, initiated on the 1–3 postoperative day. Full enteral feeding was usually reached at the seventh postoperative day.

The postoperative management was the same during the period studied. The course of postoperative ventilation was 1–3 days. Feeding started through a nasogastric tube on the first or second postoperative day. Antireflux medication was administered to all during at least the first three months postoperative period from 2008.

The primary outcome was the development of AS necessitating endoscopic dilatation. The end point of the study was 1 year after EA reconstruction.

Contrast esophagograms were performed routinely at postoperative months 1–3, 6–8, and 12 or upon clinical suspicion of stricture, that is, dysphagia, difficulty when swallowing, and/or repeated vomiting. If radiologic signs of stricture or severe dysphagia were present, examination with endoscopy was performed. AS was defined as a narrowing of the esophagus, as verified by esophagoscopy. Indication for endoscopic examination was symptoms and/or present signs of stricture on an esophagogram.

Dilatation was defined as a widening of the AS diameter as much as the caliber of a child's thumb [12]. Endoscopic dilatation involved the use of controlled radial European balloon dilators (Boston Scientific, Watertown, MA, USA) and a video endoscope (GIF-XP160; Olympus Corp, Tokyo, Japan), with patients under general anesthesia. Balloons used for dilatation were inflated with contrast during fluoroscopic imaging. If balloon contours were reduced by AS, the procedure qualified as a dilatation. If not, it was viewed as a calibration. Calibrations did not count as a dilatation.

Dilatation or calibration was performed no earlier than 3 weeks after the initial reconstruction and was repeated at intervals of 2 to 3 weeks, as needed per parent-reported symptoms or for stricture resolution on esophagogram. Adjuncts to dilatation such as local steroid injections, topical mitomycin C applications, and esophageal stents were not used during the study interval.

### 2.1. Statistical Analysis

A statistician determined that if the true probability of exposure among cases was 0.8, we needed to study nine case patients and 72 control patients to be able to reject the null hypothesis that the exposure rates for cases and controls are equal with a probability (power) of 0.8. The type I error probability associated with the test of this null hypothesis was 0.05.

Fisher's two-tailed exact test was used for comparison of dichotomous data, and the Mann–Whitney *U* test was used for comparison of continuous data. A *p* value < 0.05 was considered statistically significant.

### 2.2. Ethical Considerations

This study was executed according to the Helsinki Declaration of 1964 and the Good Clinical Practice (GCP) guidelines. The included children were registered according to the regional demands on quality register, number 01481271007173. The data were coded and deidentified. The study was approved by the Regional Ethical Review Board (registration number 2010/49). Data were anonymized before analyses were performed, and the presentation method precludes participant identification. All patients were treated per the standards of care for patients with EA at our pediatric surgical center and according to national and international guidelines.

## 3. Results

Ninety-six children were born with EA Gross type C during the study period. No patients were treated by stricture resection and second esophageal anastomosis.

In total, 72 out of 96 (75%) children with EA had any degree of CHD ranging from mild to severe. The mild types of CHD were patent ductus arteriosus (PDA) (55 patients; 76%), ventricular septal defect (VSD) (seven patients; 10%), and atrial septal defect (ASD) (three patients; 4%). The different severe CHD types are summarized in Table 1. All the CHD were diagnosed before EA repair, and all severe CHD cases were diagnosed prenatally. In two babies, the correction of EA had to be postponed until after the cardiac surgery.

**Figure 1:**
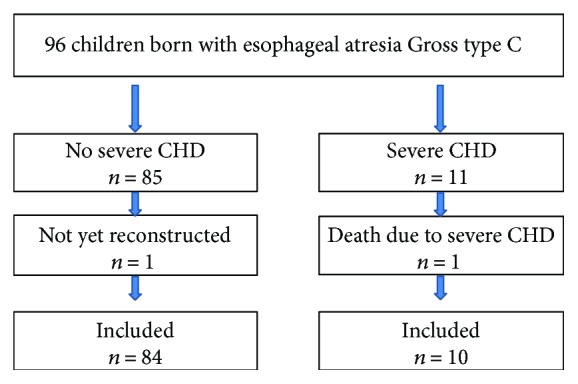
Flow chart of the study cohort including children with esophageal atresia Gross type C and congenital heart disease (CHD) born from January1996 to May 2016.

Excluded from follow-up regarding AS was one patient with EA who had not yet undergone final reconstruction at the time of follow-up due to a long gap. Out of the 95 children, who had undergone primary EA reconstruction, 11 children (12%) had severe CHD anomalies. One child died within 4 weeks after reconstruction due to concomitant complicated CHD. Then, 94 patients remained included in the follow-up of whom 10 children had severe CHD, which required cardiac surgery during the first year of life (Figure 1). The 10 children with severe CHD had their first operative interventions for CHD at a median age of 15 days (range 1 to 42 days). During the 10 surgical occasions, 19 different primary cardiac surgical interventions were performed (Table 2).

There was no difference in birth weight, gestational age, and small for gestational age or gender between the children with and without severe CHD. No child had any recurrent fistula. Among children with severe CHD, 60% required dilatation during their first year of life compared to 38% among children without CHD or mild CHD, but the difference did not reach statistical difference. Neither was there any difference in numbers of dilatations needed in each child comparing children with and without CHD (Table 3).

## 4. Discussion

In this cohort of children with EA, the presence of severe CHD did not significantly influence the need for dilatation of AS. Still, dilatations were undertaken in 60% of patients with concomitant severe CHD as compared to 38% of patients without severe CHD. To our knowledge, this is the first report describing the spectra of severe CHD concomitant to EA and their possible influence on the development of anastomotic strictures after EA reconstruction.

The frequency of severe CHD in our cohort (12%) did not differ from a previous report of a similar population with EA [12, 13]. In our study, the patients with severe CHD all underwent operative CHD intervention within the first 2 months of life, when the risk of developing AS has also been shown to be the greatest [9]. Despite this, the rates of dilatation procedures (38%/60%) were in the midrange of widely disparate reported dilatation frequencies (9–79%) [3–8]. Neither did the frequency of dilatations in children with CHD differ from dilatation frequencies reported on children with EA with unspecified frequency of CHD [9–12].

The findings in this study did not reveal any statistically increased prevalence of AS after EA reconstruction among patients with severe CHD. However, there was a tendency towards a significantly higher frequency of dilatations in patients with severe CHD, but since the sample size was small, the power became weak. Furthermore, it can only be speculated if AS is attributable to impaired vascular supply because of the underlying CHD or cardiac surgery. A common attribute to impaired vascular supply of esophagus in various CHD are, for example, hypoplastic hearth and vascular ring and coarctation. However, the hemodynamic in these CHD seems to be really different.

A confounding factor could be that 62 out of the 84 (74%) children who did not undergo surgical intervention of CHD still had minor cardiac anomalies or physiological variations such as PDA, ASD, or minor VSD. These anomalies, or their medications, might also have influenced the development of AS. However, we chose only to study the possible effects of the severe CHD types, so as to avoid divergences.

Many other causative factors for anastomotic strictures in patients with EA have been discussed before, one of them being gastroesophageal reflux (GER). However, the hypothesis that AS after EA reconstruction would be caused by GER and could be reduced by using antireflux medication as proton pump inhibitors has never been supported by any clinical evidence [9–11, 13, 14], and GER was not analyzed in this study.

Although significant differences were absent, the results suggest that children with combined EA and CHD should undergo careful observation and monitoring while awaiting cardiac surgery and after the cardiac intervention. Children with CHD often develop anorexia, vomiting, and failure to thrive, symptoms that may also be seen after EA reconstruction and as a result of AS. Thus, the combination of the two diagnoses indicates a need for closer monitoring.

Associated complication as anastomotic leak had no correlation with anastomotic stricture in this study. Recurrent TEF did not occur in this cohort.

Thus, the spectrum of congenital heart defects is heterogeneous, and there is no report about the incidence of gastroesophageal reflux in this cohort during the first year of life.

The strength of this study was that the perioperative management of all included patients was managed at the same center and was orchestrated by the same seven surgeons from the same team, with meticulous and continuous evaluations of outcomes over time. Since the center is also an NSCM in pediatric cardiac surgery and pediatric surgery for EA, the cohort is representative of children with concomitant esophageal and cardiac malformations. However, the retrospective compilation of data retrospective at first time period might constitute a limitation. In addition, the number of patients included in the cohort is small, increasing the risk of a type-two error. Another limitation is that the study focused on the first year of life and thus cannot be generalized over a longer time period, although the need for dilatations significantly decreases after the first living year [9]. Long-term follow-ups of EA in adulthood would be of interest especially in patients with both CHD and EA. In summary, the limited number of CHD patients (10), the heterogeneity of CHD, and cardiac intervention (19) are the substantial limitations of this study.

Adequate information regarding the likelihood that a child will require postoperative AS dilatation is important both for parental counseling and for planning long-term postoperative care in patients with EA. The findings in this study have been implemented in clinical work at our medical center in the way that children with combined CHD and EA have a designed observational and nutritional scheme. It is imperative that parents of patients with EA, and especially of children with concomitant severe CHD, receive information about the potential risk for anastomotic narrowing and the possible need to undergo further anesthesia for AS dilatation, particularly during the first year of life.

## 5. Conclusions

The need for AS dilatation in children with EA and concomitant severe CHD is high, although not significantly different from the children without CHD. This information is of value in parent counseling.

## Figures and Tables

**Table 1:** Concomitant severe congenital heart disease in 11 children with esophageal atresia.

Diagnosis	*n*
Ventricular septal defect: multiple (2); muscular (1); perimembranous (2); subaortic (1)	6
Atrial septal defect: secundum (5); primum (1)	6
Patent ductus arteriosus	4
Persistent left superior vena cava	2
Total anomalous pulmonary venous drainage	2
Hypoplastic left (1) or right (1) heart syndrome	2
Double outlet right ventricle	1
Coarctation of the aorta	1
Aortic stenosis	1
Vascular ring	1

**Table 2:** The primary cardiac surgical interventions performed during the first year of life in 10 children with primary reconstructed esophageal atresia (EA) and concomitant congenital heart disease (CHD).

Operative intervention	*n*
Closure of VSD with Gore-Tex patch	4
Closure of ASD by direct suturing (2) or Gore-Tex patch (1)	3
Suturing of PFD	3
Operative repair of coarctation of the aorta	2
Norwood palliation	1
Correction of TAPVR	1
Correction of DORV	1
Pulmonary artery banding	1
Atrial septectomy	1
Resection of subvalvular aortic stenosis	1
Division of vascular ring	1
Total	19

VSD: ventricular septal defect; ASD: atrial septal defect; PDA: patent ductus arteriosus; TAPVD: total anomalous pulmonary venous drainage; DORV: double outlet right ventricle.

**Table 3:** Perinatal factors and the frequency and number of dilatations of anastomotic strictures (AS) occurring within 1 year following repair of esophageal atresia (EA) in children with and without congenital heart defects (CHD).

Esophageal atresia	Without severe CHD (*n* = 84)	With severe CHD (*n* = 10)	*p* value
Gender (female/male)	29/55	4/6	0.73^∗^
Birth weight, kg	2.8 (0.8–4.1)	2.9 (1.4–4.4)	0.42^∗∗^
Gestational age, weeks	39 (26–43)	39 (30–42)	0.69^∗∗^
Small for gestational age	11 (13%)	2 (20%)	0.83^∗^
At least one dilatation of AS	32 (38%)	6 (60%)	0.31^∗^
The age at first dilatation, weeks	4	5	0.92^∗∗^
Number of AS dilatations per patient	4 (1–6)	3 (1–7)	0.91^∗∗^

Values presented as median (min–max) and as the absolute number and percentage of patients, *n* (%); ^∗^Fisher exact probability test: two-tailed; ^∗∗^Mann–Whitney *U* test.

## Data Availability

All the underlying data are available from the Department of Pediatric Surgery, Skåne University Hospital, Lund, Sweden, through the corresponding author.

## References

[1] Encinas J. L., Luis A. L., Avila L. F. (2006). Impact of preoperative diagnosis of congenital heart disease on the treatment of esophageal atresia. *Pediatric Surgery International*.

[2] Donoso F., Kassa A. M., Gustafson E., Meurling S., Lilja H. E. (2016). Outcome and management in infants with esophageal atresia—a single centre observational study. *Journal of Pediatric Surgery*.

[3] Baird R., Laberge J. M., Lévesque D. (2013). Anastomotic stricture after esophageal atresia repair: a critical review of recent literature. *European Journal of Pediatric Surgery*.

[4] Allin B., Knight M., Johnson P., Burge D., on behalf of BAPS-CASS (2014). Outcomes at one-year post anastomosis from a national cohort of infants with oesophageal atresia. *PLoS One*.

[5] Dingemann C., Dietrich J., Zeidler J. (2016). Early complications after esophageal atresia repair: analysis of a German health insurance database covering a population of 8 million. *Diseases of the Esophagus*.

[6] Landisch R. M., Foster S., Gregg D. (2017). Utilizing stricture indices to predict dilation of strictures after esophageal atresia repair. *Journal of Surgical Research*.

[7] Okata Y., Maeda K., Bitoh Y. (2016). Evaluation of the intraoperative risk factors for esophageal anastomotic complications after primary repair of esophageal atresia with tracheoesophageal fistula. *Pediatric Surgery International*.

[8] Okuyama H., Koga H., Ishimaru T. (2015). Current practice and outcomes of thoracoscopic esophageal atresia and tracheoesophageal fistula repair: a multi-institutional analysis in Japan. *Journal of Laparoendoscopic & Advanced Surgical Techniques*.

[9] Stenström P., Anderberg M., Börjesson A., Arnbjörnsson E. (2017). Dilations of anastomotic strictures over time after repair of esophageal atresia. *Pediatric Surgery International*.

[10] Hagander L., Muszynska C., Arnbjornsson E., Sandgren K. (2012). Prophylactic treatment with proton pump inhibitors in children operated on for oesophageal atresia. *European Journal of Pediatric Surgery*.

[11] Stenström P., Anderberg M., Börjesson A., Arnbjörnsson E. (2017). Prolonged use of proton pump inhibitors as stricture prophylaxis in infants with reconstructed esophageal atresia. *European Journal of Pediatric Surgery*.

[12] Sandgren K., Malmfors G. (1998). Balloon dilatation of oesophageal strictures in children. *European Journal of Pediatric Surgery*.

[13] Donoso F., Lilja H. E. (2017). Risk factors for anastomotic strictures after esophageal atresia repair: prophylactic proton pump inhibitors do not reduce the incidence of strictures. *European Journal of Pediatric Surgery*.

[14] Miyake H., Chen Y., Hock A., Seo S., Koike Y., Pierro A. (2018). Are prophylactic anti-reflux medications effective after esophageal atresia repair? Systematic review and meta-analysis. *Pediatric Surgery International*.

